# Endoscopic ultrasound-guided antegrade biliary intervention for choledocholithiasis in total gastrectomy with Roux-en-Y anatomy

**DOI:** 10.1055/a-2615-1464

**Published:** 2025-07-09

**Authors:** Boshen Lin, Zheng Zhang, Chun Li, Peng Li

**Affiliations:** 1 Beijing Friendship Hospital, Capital Medical University, Department of Gastroenterology, Beijing, China; 2 Department of Gastroenterology, Beijing Friendship Hospital, Capital Medical University, Beijing, China

Endoscopic retrograde cholangiopancreatography in patients with a history of total gastrectomy and Roux-en-Y anastomosis is challenging due to the difficulty in locating the papilla 1 2 . Meanwhile, increased intestinal motility and the considerable distance between the anastomosed limb and the left hepatic lobe also contribute to the failure in identifying a suitable puncture path during endoscopic ultrasound (EUS)-guided biliary drainage in these patients. Therefore, selecting a favorable puncture path for the left liver in the appropriate postoperative bowel is the key to successful EUS procedures.

A 77-year-old woman with a history of total gastrectomy and Roux-en-Y reconstruction for gastric cancer was admitted to our hospital with abdominal pain. Magnetic resonance imaging revealed choledocholithiasis ( Fig. 1 ). The duodenal papilla was not located on initial endoscopy despite use of a gastroscope, colonoscope, and enteroscope. Subsequently, a linear echoendoscope was inserted into the jejunum through the anastomosis. Both afferent and efferent limbs were examined to identify an optimal puncture site for accessing the intrahepatic bile duct ( Fig. 2 ). Eventually, the intrahepatic bile duct in liver segment S2, within the afferent limb, was selected. The mildly dilated left intrahepatic bile duct (approximately 0.4 cm) was accessed using a 19-gauge puncture needle ( Video 1 ). Cholangiography revealed a stone in the common bile duct (CBD). A guidewire was inserted; however, antegrade advancement into the distal CBD was challenging. Consequently, a 6-Fr cystotome was advanced over the guidewire for tract dilation. The distal CBD was successfully accessed, and the guidewire was advanced through the duodenal papilla into the duodenal lumen. A dilation balloon was used to expand the duct to 1.0 cm sequentially ( Fig. 3 ); the stone was extracted into the duodenal lumen using a retrieval balloon. Finally, a 7 Fr × 15 cm double-pigtail biliary stent was placed, with two ends in the duodenal and jejunal lumens, respectively ( Fig. 4 ).

**Fig. 1:**
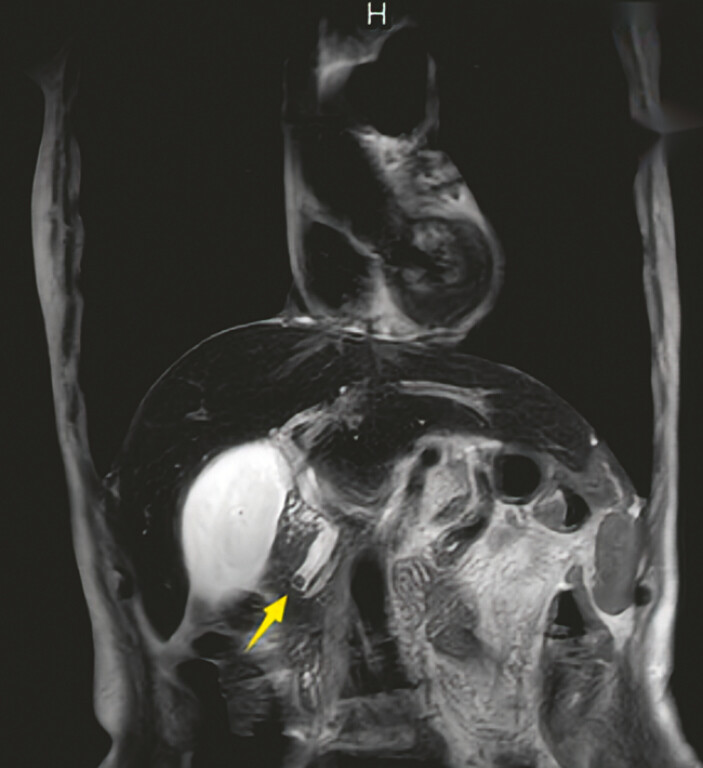
Magnetic resonance imaging revealed a stone in the common bile duct and mild dilation of the upstream duct (indicated by the yellow arrow).

**Fig. 2:**
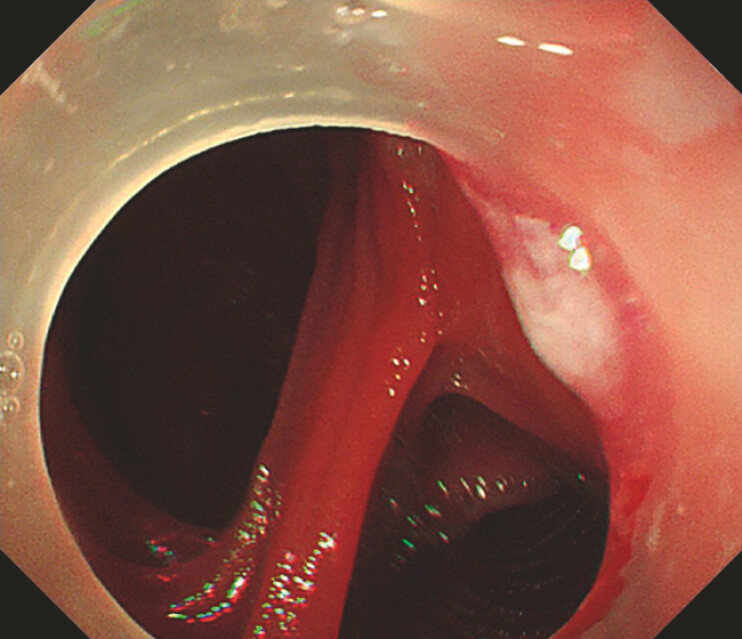
In the endoscopic view, the efferent limb is on the left, and the afferent limb is on the right.

**Fig. 3:**
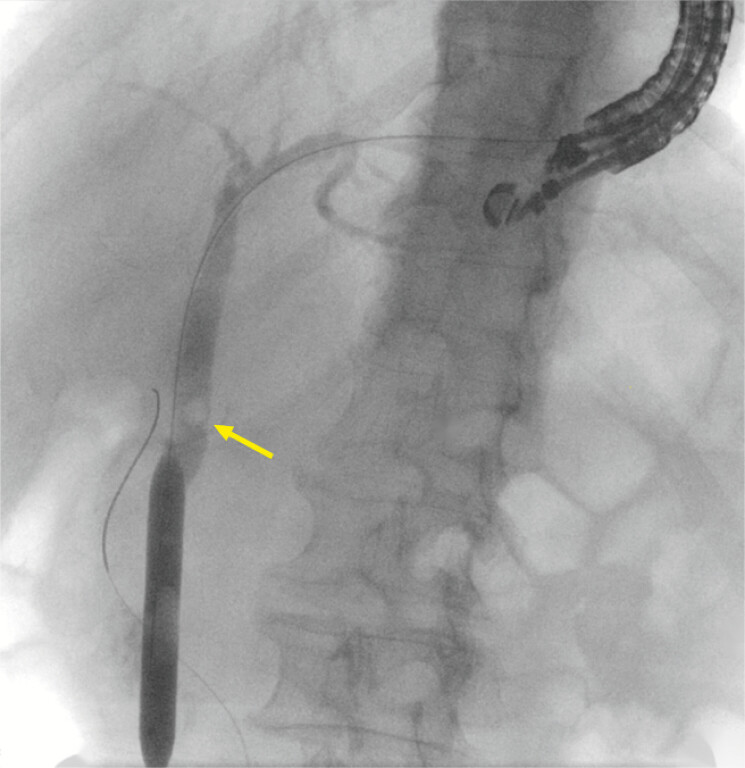
A dilation balloon was used to expand the common bile duct (CBD). The arrow points to a stone in the CBD.

**Fig. 4:**
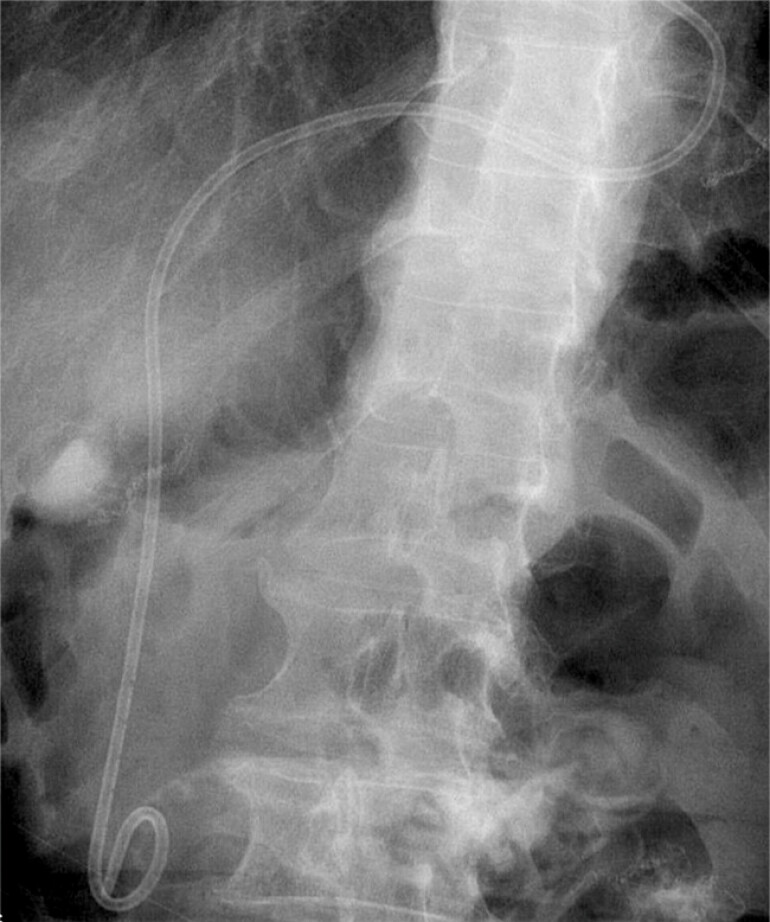
A double-pigtail biliary stent was placed, with two ends in the duodenal and jejunal lumens, respectively.

Endoscopic ultrasound-guided antegrade biliary intervention for choledocholithiasis in total gastrectomy with Roux-en-Y anatomy.Video 1

Postoperatively, the patient’s serum lipase and amylase levels remained within normal limits, whereas bilirubin levels showed a mild transient elevation that normalized within 3 days. The patient was discharged 1 week after surgery.

Endoscopy_UCTN_Code_TTT_1AS_2AD

## Notes

Endoscopy E-Videos https://eref.thieme.de/e-videos
*E-Videos* is an open access online section of the journal *Endoscopy* , reporting on interesting cases and new techniques in gastroenterological endoscopy. All papers include a high-quality video and are published with a Creative Commons CC-BY license. Endoscopy E-Videos qualify for HINARI discounts and waivers and eligibility is automatically checked during the submission process. We grant 100% waivers to articles whose corresponding authors are based in Group A countries and 50% waivers to those who are based in Group B countries as classified by Research4Life (see: https://www.research4life.org/access/eligibility/ ). This section has its own submission website at https://mc.manuscriptcentral.com/e-videos .

## References

[1] Iwashita T Yasuda I Doi S Endoscopic ultrasound-guided antegrade papillary balloon dilation for treating a common bile duct stone Dig Endosc 2013 25 89 90 10.1111/j.1443-1661.2012.01381.x 23286267

[2] Itoi T Sofuni A Tsuchiya T Endoscopic ultrasonography-guided transhepatic antegrade stone removal in patients with surgically altered anatomy: case series and technical review (with videos) J Hepatobiliary Pancreat Sci 2014 21 E86 E93 25231935 10.1002/jhbp.165

